# HMGB1/TREM2 positive feedback loop drives the development of radioresistance and immune escape of glioblastoma by regulating TLR4/Akt signaling

**DOI:** 10.1186/s12967-024-05489-w

**Published:** 2024-07-29

**Authors:** Hui Qiu, Zhiying Shao, Xin Wen, Debao Qu, Zhengyang Liu, Ziqin Chen, Xinyan Zhang, Xin Ding, Longzhen Zhang

**Affiliations:** 1 Cancer Institute, Xuzhou Medical University, Xuzhou, 221000 Jiangsu China; 2 Department of Radiation Oncology, Affiliated Hospital of Xuzhou Medical University, No. 9 Kunpeng North Road, Xuzhou, 221000 Jiangsu China

**Keywords:** TREM2, Radioresistance, Immune escape, Glioblastoma, Positive feedback loop

## Abstract

**Background:**

Radioresistance and immune escape are crucial reasons for unsatisfactory therapeutic effects of glioblastoma (GBM). Although triggering receptor expressed on myeloid cells-2 (TREM2) involved in forming immunosuppressive microenvironment, but the underlying mechanism and its roles in mediating cancer radioresistance remain unclear, moreover, the efficient delivery of drugs targeting TREM2 to GBM encounters serious challenges. Hence, this study aimed to elucidate the effect and mechanisms of targeted TREM2 silencing on reversing the radioresistance and immune escape of GBM aided by a glutathione-responsive biomimetic nanoparticle (NP) platform.

**Methods:**

Radioresistant GBM cell lines and TREM2 stable knockdown GBM cell lines were firstly established. RNA sequencing, colony formation assay, western blot, enzyme-linked immunosorbent assay and co-immunoprecipitation assay were used to detect the molecular mechanisms of TREM2 in regulating the radioresistance and immune escape of GBM. The glutathione-responsive biomimetic NP, angiopep-2 (A2)- cell membrane (CM)-NP/siTREM2/spam1, was then constructed to triply and targeted inhibit TREM2 for in vivo study. Orthotopic GBM-bearing mouse models were established to evaluate the anti-GBM effect of TREM2 inhibition, multiplex immunofluorescence assay was conducted to detect the infiltration of immune cells.

**Results:**

TREM2 was a regulator in accelerating the radioresistance and immune escape of GBM through participating in DNA damage repair and forming a positive feedback loop with high mobility group box 1 (HMGB1) to cascade the activation of Toll-like receptor 4 (TLR4)/protein kinase B (Akt) signaling. A2-CM-NP/siTREM2/spam1 was successfully synthesized with excellent passive targeting, active targeting and homologous targeting, and the in vivo results exhibited its remarkable anti-GBM therapeutic effect through promoting the infiltration of type 1 helper T cells and CD8^+^T cells, reducing the infiltration of type 2 helper T cells and regulatory T cells, repolarizing macrophages to M1-type, and decreasing the secretion of pro-tumor and immunosuppressive cytokines.

**Conclusions:**

Targeting TREM2 therapy is a promising avenue for optimizing radiotherapy and immunotherapy to improve the prognosis of GBM patients.

**Supplementary Information:**

The online version contains supplementary material available at 10.1186/s12967-024-05489-w.

## Background

Glioblastoma (GBM) is the most aggressive and intractable primary malignant brain tumor, even receiving the standard resection of tumor to the extent safely feasible and followed by radiotherapy with concurrent and adjuvant temozolomide chemotherapy, patients suffered with GBM still experience awful prognosis with short median survival (14.6 months) and low two-year survival rate (26.5%) [1–3]. Currently, the standard treatment for GBM has reached a bottleneck stage due to the existence of blood-brain barrier (BBB) and extracellular matrix barrier which limit the use of anti-GBM drugs, and enhance the susceptibility to chemoresistance and radioresistance [4–6].

Although several studies have demonstrated that immune checkpoint inhibitors (ICIs) achieve exciting clinical therapeutic efficacies in lung cancer, esophageal cancer, and other solid tumors [7–9], they brought very little therapeutic effect in GBM [10–12]. The involved mechanisms have been rigorously investigated over the past few years, and GBM microenvironment has been convincingly demonstrated as a highly immunosuppressive milieu of tumor cells and immune cells [13]. For example, GBM cells express high levels of immunosuppressive checkpoints such as programmed cell death 1-ligand 1 [14]; GBM microenvironment possesses a paucity of infiltrating T cells, especially anti-tumor T cells [15]; tumor-associated macrophages, which account for up to 30–50% of the total tumor composition, are the most abundant non-neoplastic cell types in GBM microenvironment, they can secrete immunosuppressive cytokines such as transforming growth factor-β1 (TGF-β1) and interleukin 10 (IL-10), and then further reduce the local myeloid and lymphoid immune cells [16, 17]. In addition, the radioresistant characteristic of GBM limits the release of tumor antigens to some extent, thus further aggravates the immunosuppressive tumor microenvironment (TME) [18]. Hence, the unique immunologically “cold” microenvironment of GBM should be given sufficient consideration when pursuing immune-based therapeutic strategies for GBM.

Triggering receptor expressed on myeloid cells-2 (TREM2), a transmembrane immunosuppressive receptor, was a crucial pathology-induced immune signaling pivot [19–21]. Currently, tumor cells and immune cells such as macrophages and dendritic cells with high expression of TREM2 are thought to fulfill momentous functions in avoiding immune surveillance and the resolution of latent inflammatory responses through multiple ways. Previous studies have encouragingly found that TREM2-deficiency and TREM2-blockade significantly induced preeminent anti-tumor responses in sarcoma, colorectal cancer, and mammary tumor models [22, 23]. Yan Y. et al. found that TREM2 was highly overexpressed in glioma-associated macrophages, and inhibiting TREM2 in microglia suppressed the growth and angiogenesis activity of glioma cells [24]. Peshoff MM et al. discovered that TREM2 was a vital regulator of phagocytosis in gliomas, compared with TREM2^−^ myeloid cells, TREM2^+^ cells displayed enhanced ability of tumor uptake [25]. However, whether the strategy of blocking TREM2 in GBM cells can turn the immunologically “cold” GBM into hot one and reverse the radioresistance of GBM cells, as well as the specific molecular mechanisms involved are still unclear now.

**Fig. 1:**
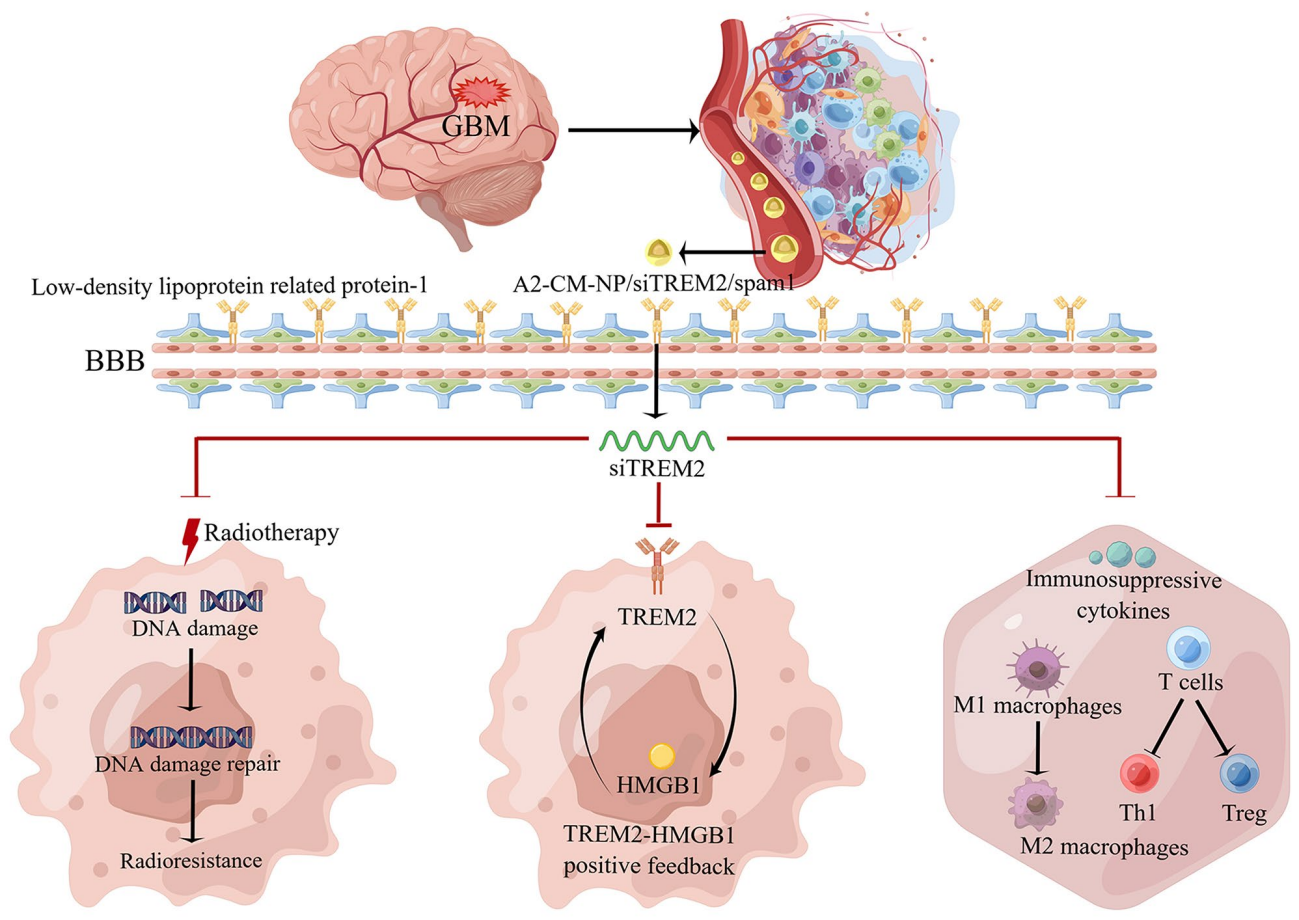
Schematic illustration of mechanism of TREM2 inhibition reversed the radioresistance and immune escape of GBM. A2-CM-NP/siTREM2/spam1 was successfully constructed with the following characteristics: passive targeting (less than 200 nm), active targeting (modified with A2), homologous targeting (coated with GBM CM vesicles), simultaneously penetrated BBB (mediated by the interaction of A2 and low-density lipoprotein related protein-1 expressed on the brain capillary endothelial cells) and degraded extracellular matrix (mediated by the co-loaded spam1 plasmid), GBM region-specific release of siTREM2 (coupled with disulfide bond). After TREM2 inhibition, the radioresistance and immune escape of GBM could be reversed by the following mechanisms: (1) directly interfering with DNA damage repair pathway; (2) inhibiting the HMGB1/TREM2 positive feedback loop; (3) restraining the secretion of immunosuppressive cytokines, the repolarization of macrophages from M1-type to M2-type, and the infiltration of pro-tumor T cells. (This figure was drawn by Figdraw.com)

Here, we illustrated that TREM2 inhibition could simultaneously solve the two intractable problems of radioresistance and immune escape in GBM through directly interfering with DNA damage repair pathway, regulating the high mobility group box 1 (HMGB1)/TREM2 positive feedback loop that cascaded the activation of Toll-like receptor 4 (TLR4)/protein kinase B(Akt) signaling pathway to decrease the secretion of pro-tumor and immunosuppressive cytokines, and inducing the polarization of immune cells towards anti-tumor phenotypes (Fig. 1). Moreover, in order to conquer the specific BBB and solid extracellular matrix barrier and efficiently silence TREM2 in GBM in vivo, TME-responsive biomimetic nanoparticles (NPs) loading the small interfering RNA (siRNA) targeting TREM2 (siTREM2), angiopepe-2(A2)-cell membrane (CM)-NP/siTREM2/spam1, were successfully synthesized and intravenously injected into orthotopic GBM-bearing mice based on the following advantages: (1) triply targeted GBM cells through passive targeting, active targeting and homologous targeting; (2) simultaneously penetrated BBB and degraded extracellular matrix barrier to facilitate more NPs accumulated in GBM region; (3) induced the GBM region-specific release of siTREM2 because of the NPs’ responsiveness to high concentration of glutathione (GSH) in GBM microenvironment. Encouragingly, the in vivo results suggested that the NPs could dramatically improve the anti-GBM responses of radiotherapy and ICI by promoting the infiltration of type 1 helper T (Th1) cells and CD8^+^T cells, but reducing the infiltration of Th2 cells and regulatory T (Treg) cells; inducing the repolarization of macrophages from M2-type to M1-type. Therefore, the reversal of radioresistance and immune escape of GBM by TREM2 inhibition is a promising avenue for optimizing radiotherapy combined with ICI treatment to improve the prognosis of GBM patients.

## Methods

### Cell lines and databases

Human GBM cell line U87MG and mouse GBM cell lines G422 and GL261 were obtained from Cell Bank, Chinese Academy of Sciences (Shanghai, China), all cells were cultured in Dulbecco modified essential medium (DMEM, HyClone, Logan, USA) supplemented with 10% fetal bovine serum (FBS, HyClone, Logan, USA) and antibiotics (100 U/mL penicillin and 100 µg/mL streptomycin, Beyotime, Shanghai, China) in a humidified atmosphere containing 5% CO_2_ at 37 °C.

The Spearman correlation of TREM2 gene with other genes were analyzed by tumor immune estimation resource 2.0 (TIMER 2.0) database (http://timer.comp-genomics.org/). The differentially expressed genes (DEGs) between GBM tissues with higher TREM2 expression and those with lower TREM2 expression was analyzed by gene expression omnibus database (https://www.ncbi.nlm.nih.gov/geo/), then Kyoto encyclopedia of genes and genomes (KEGG) and gene ontology (GO) analysis were performed with the significant DEGs. A protein-protein interaction network for TREM2 was designed with the help of search tool for recurring instances of neighboring genes (STRING) database (https://cn.string-db.org/).

### Establishment of radioresistant GBM cells

According to the calculation formula of bioequivalent dose (BED), BED = nd [1 + d/(α/β)] (n is the fractional number, d is the fractional dose, α/β = 10), combined with the total irradiation (IR) dose of 60 Gy (2 Gy per fraction) given in clinical treatment of GBM patients [2], U87MG and GL261 cells were exposed to 50 Gy in 10 fractions with a dose of 5 Gy per fraction using an X-RAD 225XL biological irradiator (Precision X-Ray Inc., Madison, USA) to establish radioresistant U87MG (U87MG-R) and radioresistant GL261 (GL261-R) cells.

After radiotherapy, clone formation assay was used to calculate sensitizing enhancement ratio (SER) to determine whether U87MG-R and GL261-R cells were successfully established, and western blot was used to detect the expression levels of key proteins involved in DNA damage repair pathway in U87MG-R and GL261-R cells.

### Colony formation assay

Cells were seeded in culture dishes at an appropriate density and cultured until the appearance of adherent growth, then cells were treated with 0 Gy, 2 Gy, 4 Gy, 6 Gy, and 8 Gy of X-ray, respectively. The medium was then changed every 3 days until cell clones (each clone contains more than 50 cells) were formed. Cell clones were washed with phosphate buffer solution (PBS; VICMED, Xuzhou, China) for 3 times, fixed with 4% paraformaldehyde (VICMED, Xuzhou, China) for 15 min and visualized by staining with crystal violet (Beyotime, Shanghai, China). After counting the clone number, cell survival curves were fitted by GraphPad Prism software 8.0 based on signal-hit multitarget model and linear-quadratic model, and several parameters including surviving fraction at 2 Gy (SF2), D0, Dq, α, β and SER_D0_ (SER_D0_=D0_control group_/D0_experimental group_) were calculated to evaluate the radiosensitivity.

### Western blot

Equal amount of total protein extracted from cells was separated by 10% sodium dodecyl sulphate-polyacrylamide gel electrophoresis (SDS-PAGE, New Cell & Molecular Biotech., Suzhou, China), separated protein bands were transferred to polyvinylidene fluoride (PVDF) membranes (Millipore, Boston, USA), which were then blocked with 5% skim milk (VICMED, Xuzhou, China) for 1 h at room temperature. The PVDF membranes were immunoblotted with primary antibodies against TREM2 (Proteintech, Wuhan, China; Cell Signaling Technology, Boston, USA), DNA-dependent protein kinase catalytic subunit (DNA-PKcs, Proteintech, Wuhan, China), KU70 (Cell Signaling Technology, Boston, USA), KU80 (Cell Signaling Technology, Boston, USA), γ-H_2_AX (Cell Signaling Technology, Boston, USA), poly ADP-ribose polymerase 1 (PARP1, Proteintech, Wuhan, China), TLR4 (Proteintech, Wuhan, China), HMGB1 (Proteintech, Wuhan, China), Akt (Proteintech, Wuhan, China), phospho-Akt (Ser473) (Proteintech, Wuhan, China), ERK1/2 (ABclonal, Wuhan, China), phospho-ERK1(202/Y204) + ERK2(T185/Y187) (phospho-ERK1/2, ABclonal, Wuhan, China), T cell immunoglobulin domain and mucin domain-3 (TIM-3, Proteintech, Wuhan, China), CD68 (Proteintech, Wuhan, China), CD80 (Proteintech, Wuhan, China), CD206 (Proteintech, Wuhan, China), and GAPDH (Proteintech, Wuhan, China) overnight at 4 °C, the antibodies were diluted according to instructions. After washing, the PVDF membranes were incubated with horseradish peroxidase-conjugated secondary antibodies (1:10000; VICMED, Xuzhou, China) at room temperature for 1 h. The immunoblotted membranes were then washed with washing buffer for 5 times and visualized with enhanced chemiluminescence kit (New Cell & Molecular Biotech., Suzhou, China) and chemiluminescence imaging system (Tanon, Shanghai, China).

### Establishment of stable TREM2 knockdown (TREM2-KD) GBM cell lines

The concentrated enhanced green fluorescent protein (EGFP)-labeled lentivirus solution expressing short hairpin RNA (shRNA) against TREM2 and negative control (NC) shRNA was constructed and packaged by OBiO Technology Corp.,Ltd. (Shanghai, China). U87MG, GL261 and G422 cells were seeded in 6-well plates and infected with the aforementioned lentivirus solution, 6 h later, the cultured medium was replaced with fresh DMEM containing 10% FBS. Cells were cultured for another 72 h and selected by puromycin (0.4 µg/mL) to establish U87MG-NC, U87MG^TREM2 − KD^, GL261-NC, GL261^TREM2 − KD^ and G422^TREM2 − KD^ cell lines. The infection efficiency was observed by fluorescence microscopy, and the KD efficiency of TREM2 was detected by western blot.

### RNA sequencing

Total RNAs were extracted from GBM-NC and GBM^TREM2−KD^ cells using Trizol reagent (Invitrogen, Carlsbad, USA) according to the manufacturer’s instructions and submitted to Biotechnology Corporation (Shanghai, China). The paired-end sequencing was performed on the Illumina HiSeq platform according to the vendor’s recommended procedure. DEGs with significance were identified as those with *P* < 0.05 and fold change of ≥ 2.0 or ≤ − 2.0. GO and KEGG pathway enrichment analyses were then performed with the significant DEGs using ggplot2 system.

### Enzyme-linked immunosorbent assay (ELISA)

Cell culture medium and peripheral blood serum samples of mice were collected and centrifuged (1000×g) at 4℃ for 20 min, then the supernatant was retained for ELISA assay to detect the concentrations of soluble TREM2 (sTREM2, ELISA kit, Lianke bio., Hangzhou, China), HMGB1 (ELISA kit, Biorbyt, Cambridge, United Kingdom), IL-6 (ELISA kit, Elabscience, Wuhan, China), IL-10 (ELISA kit, Elabscience, Wuhan, China), TGF-β1 (ELISA kit, Elabscience, Wuhan, China), and interferon-gamma (IFN-γ) according to the manufacturers’ instructions.

### Co-immunoprecipitation (Co-IP) assay

Equal cell lysates (1 mg) from harvested cells were incubated with anti-TREM2 primary antibody (1 µg) or anti-IgG antibody (1 µg) at 4℃ overnight, then 25µL protein A/G PLUS-agarose beads (Santa Cruz Biotechnology, Texas, USA) were added to the lysates and incubated for 4 h at 4 °C. After washing with PBS, the bead-antibody complexes were suspended in 2×SDS loading buffer and boiled for 10 min. Subsequently, the immunoprecipitants were subjected to western blot.

### siTREM2 transfection

Six siTREM2s and one siNC were synthesized by Sangon Biotech Co., Ltd. (Shanghai, China), and their sequences were shown in Table S1. The siRNAs were transfected into GBM cell lines by using Lipo6000™ transfection reagent (Beyotime, Shanghai, China) according to the manufacturer’s instruction. 72 h later, the expression level of TREM2 was detected by western blot.

### Construction of GSH-responsive biomimetic A2-CM-NP/siTREM2/spam1

#### A2-NP preparation

A2, 1,2-dioleoyl-3-trimethylammonium propane (DOTAP), dioleoylphosphatidylethanolamine (DOPE) and 1,2-distearoyl-sn-glycero-3-phosphoethanolamine (DSPE)-disulfide bond (SS)-polyethylene glycol 2000 (PEG2000)-maleimide (Mal) (Ruixi Biological Technology, Xi’an, China) were completely solubilized in absolute ethanol (Sinopharm Chemical Reagent, Shanghai, China), respectively. A2 was conjugated with DSPE-SS-PEG2000-Mal at the molar ratio was 1:1, the reaction mixture was stirred at room temperature for 1 h to obtain DSPE-SS-PEG-A2. DOTAP and DOPE solution were then added to DSPE-SS-PEG-A2 at the molar ratio was DOTAP: DOPE: DSPE-SS-PEG2000-Mal: A2 = 6:3:1:1 and kept vortex oscillation for 2 min. After that, the mixture was dripped into diethyl pyrocarbonate (DEPC)-treated water and transferred into a dialysis bag, which was then placed in a beaker of DEPC-treated water and gently stirred for 3 h at room temperature. After dialysis, the mixture in the dialysis bag was recovered as the synthesized A2-NP and stored at room temperature.

#### Construction of A2-NP/siTREM2/spam1

According to the molar ratio of N/P, appropriate amounts of A2-NP, siTREM2 and spam1 plasmid (GenePharma, Suzhou, China) were added in a centrifuge tube respectively, then filled the A2-NP tube and siTREM2/spam1 plasmid tube to equal volume with DEPC-treated water. Subsequently, the siTREM2/spam1 plasmid solution was gently added to the A2-NP solution, after gently mixing, the mixture was stayed at room temperature for 15 min to construct A2-NP/siTREM2/spam1.

#### Extraction of CM vesicles

The CM vesicles of GBM cells were extracted with the use of membrane and cytosol protein extraction kit (Beyotime, Shanghai, China) according to the manufacturer’s instruction. Briefly, the harvested GBM cells were repeatedly frozen in liquid nitrogen and thawed at room temperature until the degree of cell fragmentation was greater than 70%. The cell debris suspension was centrifuged at 700×g for 10 min at 4 °C to remove the cell nuclei and intact cells. The supernatant was then centrifuged at 14,000×g for 30 min at 4 °C to collect the precipitation containing CM vesicles by discarding the supernatant. Finally, the CM vesicles were freeze-dried with liquid nitrogen and stored at -80℃ for later use.

#### Preparation of A2-CM-NP/siTREM2/spam1

For membrane coating, appropriate amount of freeze-dried CM vesicles suspended in sterilized double distilled water (ddH_2_O) were mixed with A2-DSPE-SS-PEG2000 and A2-NP/siTREM2/spam1 at the weight ratio of 1:1 (CM vesicles : A2-NP). After vortex stirring for 10 min, the mixture was continuously extruded through the liposome extruders with reduced pore size (400 nm and 200 nm) to obtain A2-CM-NP/siTREM2/spam1.

### Characterization of A2-CM-NP/siTREM2/spam1

The encapsulation efficiencies of A2-NP/siTREM2/spam1 were evaluated by agarose gel electrophoresis. 2% agarose gel was prepared and samples of siTREM2, spam1 plasmid and A2-NP/siTREM2/spam1 constructed with different N/P ratios (1:1, 6:1, 12:1, 18:1, 24:1, 30:1 and 36:1) were separately added into the bottom of wells. The electrophoretic voltage was 120 V and the electrophoresis time was 15 min, then visualized the RNA and DNA fragments by using the ultraviolet gel image analysis system (Tanon, Shanghai, China).

The coating of CM vesicles onto NPs was detected by SDS-PAGE. Briefly, whole cells, CM vesicles and A2-CM-NP/siTREM2/spam1 were lysed in RIPA lysis buffer (Beyotime, Shanghai, China) containing protein inhibitor cocktail (MedChemExpress, New Jersey, USA) and then subjected to SDS-PAGE. After electrophoresis, the gel was stained with Coomassie blue staining solution (Beyotime, Shanghai, China) for 1 h at room temperature, then soaking and slowly shaking the gel in destaining solution at room temperature until the protein bands are clearly visible. The particle size and surface charge (zeta potential) of A2-NP, A2-NP/siTREM2/spam1 and A2-CM-NP/siTREM2/spam1 were determined by dynamic light scattering using a Malvern Zetasizer (Worcestershire, United Kingdom) at 25℃.

### GBM targeting efficiency of A2-CM-NP/siTREM2/spam1

#### In vitro active GBM targeting

GL261 cells were seeded in 6-well plates and divided into three groups including Cy3-siRNA group, A2-NP/cy3-siRNA group and A2-CM-NP/cy3-siRNA group. After overnight culture, Cy3-siRNA (100pmol), A2-NP/cy3-siRNA (containing 100pmol cy3-siRNA) and A2-CM-NP/cy3-siRNA (constructed with GL261 CM vesicles, containing 100pmol cy3-siRNA) were added to the corresponding well respectively. After incubating for 6 h, cells were fixed with 4% paraformaldehyde for 10 min and then stained with Hoechst 33,342 (Thermo Fisher Scientific, Waltham, USA) for 10 min at room temperature. Cell imaging was performed on the Olympus IX73 fluorescence microscope (Tokyo, Japan), the intensity of red fluorescence in each group was then analyzed with ImageJ software.

#### In vitro homologous GBM targeting

GL261, G422 and U87MG cells were seeded in 6-well plates and cultured overnight, A2-CM-NP/cy3-siRNA (constructed with GL261 CM vesicles, containing 100pmol cy3-siRNA) were then added to every well respectively. 6 h later, cells were fixed with 4% paraformaldehyde for 10 min and stained with Hoechst 33,342 for 10 min at room temperature. Finally, cells were imaged under the Olympus IX73 fluorescence microscope and the intensity of red fluorescence in each group was analyzed with ImageJ software.

#### Biodistribution of A2-CM-NP/siTREM2/spam1 in mice bearing orthotopic GBM

C57BL/6 mice (male, 6 ~ 8 weeks) were purchased from Gempharmatech Co., Ltd. (Nanjing, China). 8 × 10^5^ luciferase (luc)-labeled GL261 cells were suspended in 10µL PBS and injected into the right striatum of mouse to establish an orthotopic GBM-bearing mouse model. After 7 days, the orthotopic GBM-bearing mouse model was successfully established by measuring the fluorescence in brain through intraperitoneal injection of D-luciferin (VICMED, Xuzhou, China) and imaging with a small animal live imaging system (Berthold, Bad Wildbad, Germany).

PBS, A2-NP/cy3-siRNA, A2-CM-NP/cy3-siRNA (synthesized with GL261 CM vesicles) and A2-CM-NP/cy3-siRNA/spam1 ((synthesized with GL261 CM vesicles) were respectively injected into orthotopic GBM-bearing mice through tail vein, the dosage of cy3-siRNA and spam1 plasmid was 0.2nmol/g (body weight) and 1 µg/g (body weight), respectively. 24 h later, the mice were euthanized by excessive carbon dioxide inhalation, their brain, heart, liver, spleen, lungs and kidneys were collected, the distribution of red fluorescence (cy3) in the above tissues were then detected by a small animal live imaging system.

### Antitumor efficacy of TREM2 inhibition in orthotopic GBM-bearing mouse model

7 days after the implantation of 8 × 10^5^ luc-labeled GL261 cells to the right striatum of C57BL/6 mice or 8 × 10^5^ luc-labeled G422 cells to the right striatum of Kunming mice (Vital River Laboratory Animal Technology Co., Ltd., Beijing, China), the mice were anesthetized with isoflurane and intraperitoneally injected with D-luciferin to detected the tumorigenesis using a small animal live imaging system. Subsequently, the orthotopic GBM-bearing mice were randomly divided into the following 8 groups: control group, IR group, programmed cell death protein 1 (PD-1) inhibitor group, A2-CM-NP/siTREM2/spam1 group, IR + PD-1 inhibitor group, IR + A2-CM-NP/siTREM2/spam1 group, PD-1 inhibitor + A2-CM-NP/siTREM2/spam1 group, and IR + PD-1 inhibitor + A2-CM-NP/siTREM2/spam1 group (*n* = 8), whole brain radiotherapy (3 Gy/fraction/days×3 days) was used with the rest parts of body were shielded with lead blocks, PD-1 inhibitor (200 µg/mouse/day×3days) and A2-CM-NP/siTREM2/spam1 (siTREM2: 0.2nmol/g body weight/day×1 day; spam1: 1 µg/g body weight/day×1 day) were administered through tail vein. On days 14 and 21, the tumor-bearing mice were fluorescently imaged again. The body weights of these mice were weighed every 3 days and their overall survival (OS) times were recorded until the emergence of observation endpoint defined as natural death, 20% weight loss and abnormal central neurological symptoms, then the Kaplan-Meier survival curves were plotted.

### Terminal deoxynucleotidyl transferase-mediated dUTP-biotin nick end labeling (TUNEL) assay

The apoptosis rates in GBM tissues were detected using the CoraLite^®^594 TUNEL assay apoptosis detection kit (Proteintech, Wuhan, China) according to the manufacturer’s instruction, and the nucleus were stained with 4’,6-diamidino-2-phenylindole (DAPI, Beyotime, Shanghai, China) for 5 min. The apoptotic cells were labelled with red fluorescence and visualized by the Olympus IX73 fluorescence microscope.

### Multiplex immunofluorescence assay

On the seventh day after treatment, three orthotopic GBM-bearing mice in each group were euthanized by excessive carbon dioxide inhalation, and their brain tissues were collected and fixed with 4% paraformaldehyde for 48 h to prepare frozen sections. After rewarming at room temperature, the sections were immersed and boiled in citrate buffer (0.01 M, pH = 6.0, Servicebio, Wuhan, China) to complete antigen retrieval, 3% hydrogen peroxide solution (Caoshanhu, Nanchang, China) was then added to each sample to block endogenous peroxidase. Next, the sections were blocked with 5% bovine serum albumin for 60 min. Primary antibodies including anti-CD3 (1:200, Proteintech, Wuhan, China), CoraLite^®^647 anti-CD4 (1:500, Proteintech, Wuhan, China), CoraLite^®^647 anti-CD8a (1:500, Proteintech, Wuhan, China), anti-GATA3 (1:200, Proteintech, Wuhan, China), anti-T-bet (1:200, Proteintech, Wuhan, China), anti-Foxp3 (1:200, Proteintech, Wuhan, China), anti-CD68 (1:1000, Proteintech, Wuhan, China), anti-CD206 (1:1000, Proteintech, Wuhan, China), and anti-CD80 (1:200, Proteintech, Wuhan, China) were incubated with the samples overnight at 4 °C. Then CoraLite488-conjugated goat anti-rabbit IgG (1:500, Proteintech, Wuhan, China) and Alexa Fluor555-conjugated goat anti-mouse IgG (1:500, Proteintech, Wuhan, China) were added and incubated with sections at room temperature for 45 min in the dark. Finally, the sections were stained with DAPI for 5 min at room temperature and covered with coverslips. Fluorescence images were captured by the Olympus BX53 fluorescence microscope (Tokyo, Japan), the fluorescence intensity of each group was then analyzed with ImageJ software.

### Hematoxylin and eosin (H&E) staining

On the seventh day after treatment, three orthotopic GBM-bearing mice in each group were sacrificed, the main organs including heart, liver, spleen, lungs and kidneys were collected and fixed with 4% paraformaldehyde for 48 h to prepare paraffin sections. After baking at 60 °C for 3 h, the sections were then dewaxed with xylene for 10 min, hydrated with gradient alcohol and ddH_2_O for 5 min every time. Hematoxylin were added to the sections and stained the nucleus for 6 min, the samples were differentiated with acid alcohol differentiation solution and stained with eosin for 1 min subsequently. Then the dehydrated sections were sealed with neutral gum (Sinopharm, Suzhou, China) and imaged under a light microscope (Olympus, Tokyo, Japan).

### Statistical analysis

All experiments were independently repeated at least three times, and data are presented as mean ± standard deviation (SD). Statistical analysis was performed using GraphPad Prism 8.0 (GraphPad Software, San Diego, USA). Spearman correlation analysis was used to calculate the correlation between two gene expression levels, two-tailed Student’s *t*-test was used for comparisons of cytokine secretion level and fluorescence intensity between two groups, and the Kaplan-Meier survival curves and Log-rank test were used to compare the survival times of mice. *P* < 0.05 were considered statistically significant.

## Results

### TREM2 was notably overexpressed in radioresistant GBM cells

To investigate the role of TREM2 in GBM radioresistance, U87MG-R and GL261-R cell lines were firstly established according to the schematic illustration shown in Fig. S1 A. The result of colony formation assay showed that the plating efficiencies of U87MG-R and GL261-R cells were significantly increased compared with U87MG and GL261 cells; moreover, the survival curves of U87MG-R and GL261-R cells descended more slowly than those of U87MG and GL261 cells (Fig. S1 B, C). After calculating the parameters related to radiosensitization, the SER_D0_ of U87MG-R and GL261-R cells was 0.819 and 0.805, respectively (Table S2), suggesting the successful establishment of radioresistant cell lines, U87MG-R and GL261-R.

Furthermore, the result shown in Fig. S1 D revealed that the expression level of DNA damage marker γ-H_2_AX in U87MG-R and GL261-R cells elevated more tardily at 2, 4, 6, 8 and 10 h after radiotherapy (2 Gy) than that in U87MG and GL261 cells, indicating conventional radiotherapy was not enough to cause DNA damage in U87MG-R and GL261-R cells, which again demonstrated that radioresistant GBM cell lines had been successfully established and could be used in subsequent experiments. We then detected the expression level of TREM2 in U87MG-R and GL261-R cells and found that, compared with U87MG and GL261 cells, U87MG-R and GL261-R cells expressed higher TREM2 protein with better stability (Fig. S1 E).

### TREM2 inhibition significantly enhanced the radiosensitivity of GBM cells

**Fig. 2:**
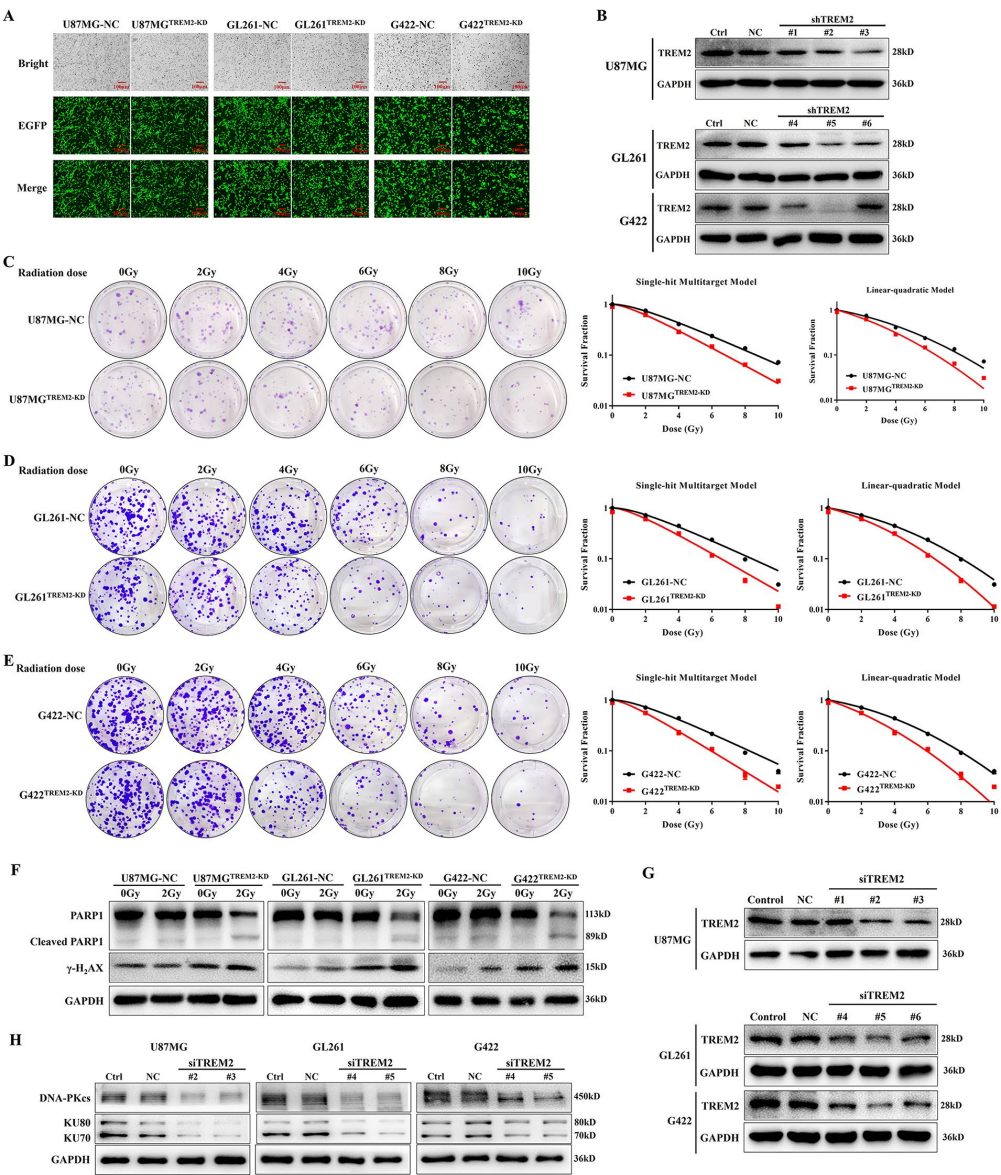
TREM2 inhibition remarkably enhanced the radiosensitivity of GBM through interfering with DNA damage repair pathway. (**A**) The infected efficiencies of EGFP-labeled lentivirus expressing TREM2-shRNA and NC-shRNA in GBM cells were observed under fluorescence microscope (scale bar: 100 μm); (**B**) The expression of TREM2 in GBM cells after TREM2 knockdown were detected by western blot; (**C**)-(**E**) The colony formation of GBM-NC cells and GBM^TREM2−KD^ cells treated with radiotherapy at different doses, and the survival curves were fitted based on signal-hit multitarget model and linear-quadratic model, respectively, data are presented as mean ± SD, *n* = 3; **(F)** The expression of apoptotic marker PARP1 and cleaved PARP1, DNA damage marker γ-H_2_AX in GBM-NC and GBM^TREM2−KD^ cells 24 h after exposing to radiotherapy (2 Gy) were detected by western blot; (**G**) The expression of TREM2 in GBM cells 48 h after TREM2 silencing with different siTREM2s were detected by western blot; (**H**) The expression of DNA-PKcs, KU70 and KU80 in GBM cells 48 h after TREM2 silencing were detected by western blot

GBM^TREM2−KD^ and paired GBM-NC cell lines were then successfully established (Fig. 2A, B) to detect the effect of TREM2 silencing on the radiosensitivity of GBM cells. As shown in Fig. 2C–E, the plating efficiencies of U87MG^TREM2 − KD^, GL261^TREM2 − KD^ and G422^TREM2 − KD^ cells were obviously decreased than those of U87MG-NC, GL261-NC and G422-NC cells, respectively. The survival fractions of U87MG^TREM2 − KD^, GL261^TREM2 − KD^ and G422^TREM2 − KD^ cells also distinctly reduced than those of U87MG-NC, GL261-NC and G422-NC cells. Based on the single-hit multitarget model and linear-quadratic model, the parameters related to radiosensitization were calculated and shown in Table S3 demonstrated that TREM2 inhibition prominently improved the radiosensitivity of U87MG, GL261 and G422 cells with the SER_D0_ was 1.223, 1.244 and 1.310, respectively.

Subsequently, GBM^TREM2−KD^ and paired GBM-NC cells were exposed to radiotherapy at the dose of 2 Gy which was commonly used in clinical practice, and we found that the expression of PARP1 in GBM^TREM2−KD^ cells was significantly decreased, while the expression of cleaved PARP1 and γ-H_2_AX were significantly increased (Fig. 2F), suggesting radiotherapy was more likely to cause DNA damage in GBM^TREM2−KD^ cells than GBM-NC cells. Several siTREM2s were then synthesized and evaluated, siTREM2#2 and siTREM2#3 were selected to transfected into U87MG cells, siTREM2#4 and siTREM2#5 were selected to transfected into GL261 and G422 cells (Fig. 2G) to detect the effect of TREM2 inhibition on the expression of key proteins involved in DNA damage repair pathway including DNA-PKcs, KU70 and KU80. The results shown in Fig. 2H revealed that TREM2 silencing clearly reduced the expression of DNA-PKcs, KU70 and KU80, indicating that TREM2 inhibition directly interfered with the DNA damage repair pathway in GBM cells.

### TREM2 was involved in shaping the immunosuppressive GBM microenvironment

**Fig. 3:**
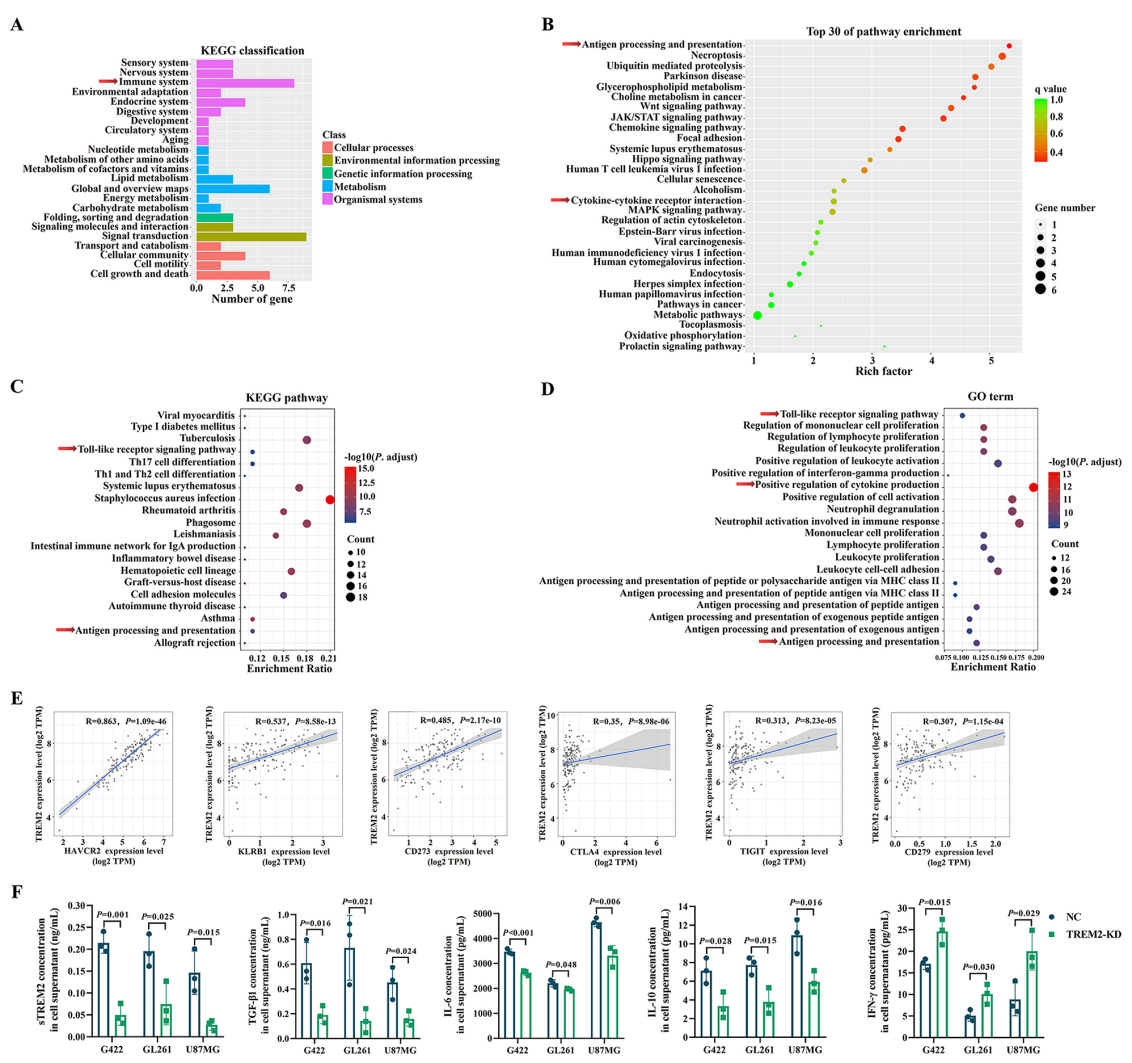
TREM2 was involved in remodeling the immunosuppressive microenvironment of GBM. (**A**)-(**B**) The KEGG classification and the top 30 of pathway enrichment of DEGs between GBM-NC cells and GBM^TREM2−KD^ cells detected by RNA sequencing; (**C**)-(**D**) The KEGG pathway enrichment and GO term analysis of DEGs between GBM tissues with low TREM2 expression and those with high TREM2 expression obtained from TCGA database; (**E**) The Spearman correlation scatterplots of TREM2 and HAVCR2, KLRB1, CD273, CTLA4, TIGIT, and CD279 genes in GBM were analyzed by TIMER2.0 database; (**F**) The effect of TREM2 inhibition on the secretion of sTREM2, TGF-β1, IL-6, IL-10 and IFN-γ from U87MG-NC, U87MG^TREM2 − KD^, GL261-NC, GL261^TREM2 − KD^, G422-NC and G422^TREM2 − KD^ cells were detected by ELISA assay, data are presented as mean ± SD, *n* = 3

To explore the specific molecular mechanism of TREM2 regulating the development of radioresistance in GBM cells, total RNAs extracted from GBM-NC cells and GBM^TREM2−KD^ cells were subjected to RNA sequencing. The results indicated that one of the crucial functions of DEGs was to regulate the immune system (Fig. 3A), and the top 30 of pathway enrichment including antigen processing and presentation, cytokine-cytokine receptor interaction (Fig. 3B). KEGG and GO analysis of data from the cancer genome atlas program (TCGA) database also suggested that the DEGs were involved in antigen processing and presentation, positive regulation of cytokine production (Fig. 3C, D).

The spearman correlations between TREM2 gene and immunosuppressive checkpoint-related genes in GBM microenvironment based on TCGA database were further analyzed, and the results shown in Fig. 3E suggested that TREM2 was positively correlated with HAVCR2, KLRB1, CD273, CTLA4, TIGIT and CD279, and the correlation between TREM2 and HAVCR2 which encoding TIM-3 protein was strongest with the Spearman’s rank correlation coefficient of 0.863.

In terms of cytokine production, the analysis results based on TIMER2.0 database suggested that TREM2 expression was positively associated with the levels of cytokines which promoted the development of cancers and triggered the formation of immunosuppressive TME including IL-6, IL-10, TGF-β1, CCL2, CCL3, CCL4, CCL5, CCL7, CCL8, CXCL2 and CXCL9, while negatively correlated with anti-tumor cytokine IL-13 production (Fig. S2). We further demonstrated that, besides sTREM2, the levels of TGF-β1, IL-6 and IL-10 secreted by GBM^TREM2−KD^ cells were significantly lower than those of GBM-NC cells, while the level of IFN-γ was obviously increased (Fig. 3F). The above results signified that TREM2 participated in remodeling the immunosuppressive GBM microenvironment, and the related in-depth molecular mechanism was worthy of further study.

### HMGB1/TREM2 positive feedback loop exhibited cascade regulation of TLR4/Akt signaling pathway

**Fig. 4:**
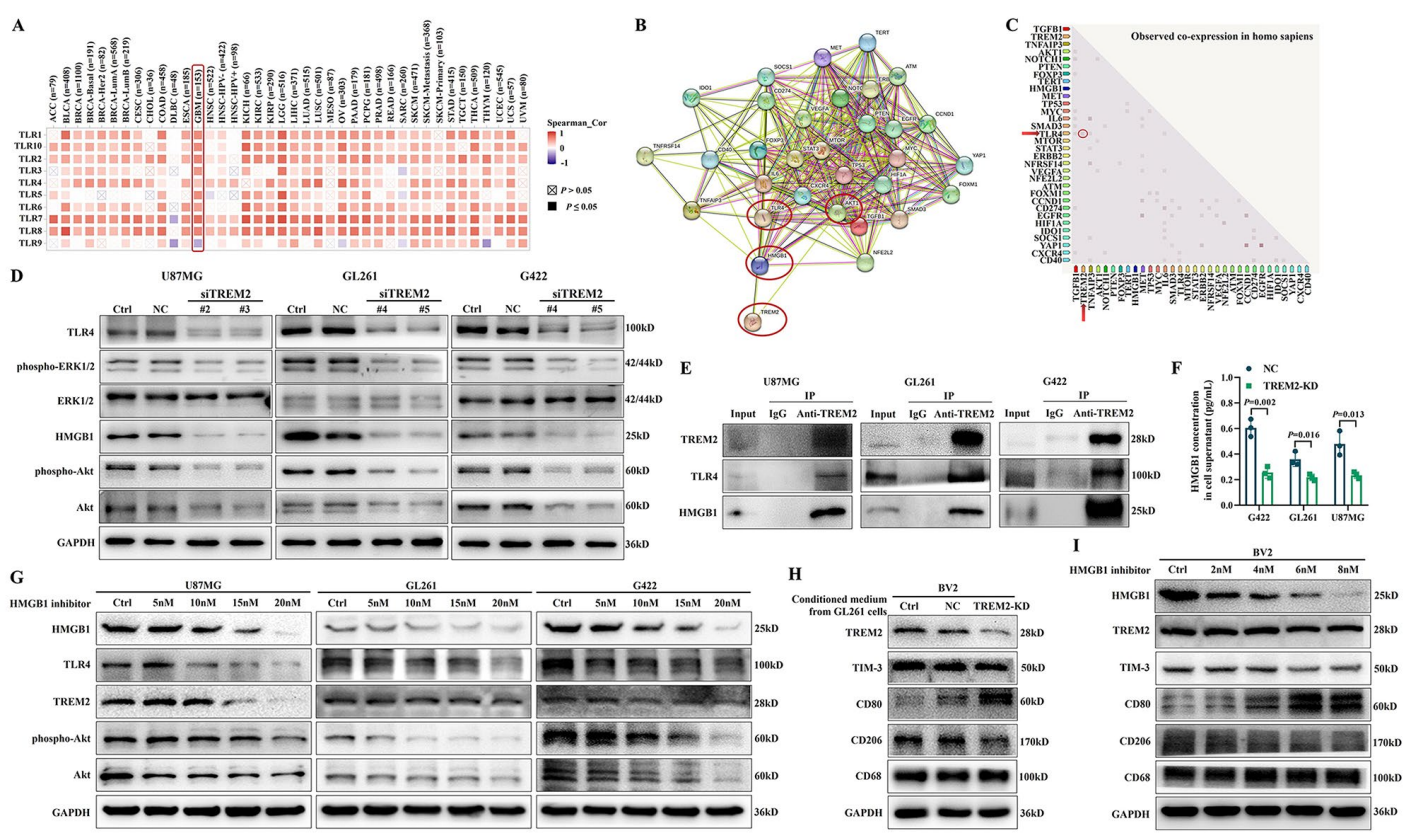
The positive feedback loop between HMGB1 and TREM2 cascade the regulation of TLR4/Akt signaling pathway. (**A**) The Spearman correlation heat map of TREM2 and TLR genes (TLR1 ~ TLR10) analyzed by TIMER2.0 database; The protein-protein interaction network (**B**) and the co-expression relationship (**C**) of TREM2 and genes related to the radioresistance and immune escape of GBM were analyzed by STRING database; (**D**) The expression of TLR4, phosphor-ERK1/2, ERK1/2, HMGB1, phospho-Akt and Akt in GBM cells 48 h after TREM2 silencing were detected by western blot; (**E**) The mutual binding between TREM2 and TLR4, HMGB1 were detected by Co-IP assay; (**F**) The effect of TREM2 inhibition on the secretion of HMGB1 from GBM cells was detected by ELISA assay, data are presented as mean ± SD, *n* = 3; (**G**) The expression of HMGB1, TLR4, TREM2, phospho-Akt and Akt in GBM cells after treatment with increased concentration (0nM, 5nM, 10nM, 15nM, 20nM) of HMGB1 inhibitor for 24 h were detected by western blot; (**H**) The expression of TREM2, TIM-3, CD80, CD206 and CD68 in BV2 cells cultured with the conditioned culture supernatant of GL261, GL261-NC and GL261^TREM2 − KD^ cells, respectively, for 48 h were detected by western blot; (**I**) The expression of HMGB1, TREM2, TIM-3, CD80, CD206 and CD68 in BV2 cells after treatment with increased concentration (0nM, 2nM, 4nM, 6nM, 8nM) of HMGB1 inhibitor for 24 h were detected by western blot

Since TREM2 was also closely associated with Toll-like receptor (TLR) signaling pathway (Fig. 3C, D), the expression correlation between TREM2 and TLRs were subsequently analyzed utilizing the data from TCGA database. As shown in Fig. 4A, in GBM microenvironment, there were significant positive correlations between TREM2 and TLR1 ~ TLR10, except TLR9. In order to further explore the molecular mechanism of TREM2 in regulating GBM radioresistance and immune escape, the top 20 genes associated with the radioresistance and immune escape of GBM were then unmasked through the National Center for Biotechnology Information website, respectively (Table S4). Among these genes, EGFR, STAT3, TP53, PTEN, VEGFA, IL-6, TGF-β1, MYC and HMGB1 were significantly correlated with both radioresistance and immune escape, and TLR4 was correlated with the immune escape of GBM. In addition, the result of protein-protein interaction network of TREM2 and these genes indicated that TREM2 had the regulatory relationship with HMGB1, TLR4 and Akt (Fig. 4B), further analysis of co-expression showed that there was only a co-expression relationship between TREM2 and TLR4 (Fig. 4C).

Because TLR4 is one of HMGB1 receptors, and TLR4 could regulate phosphatidylinositol 3 kinase (PI3K)/Akt signaling pathway and induce cytokine secretion through mitogen-activated protein kinase signaling [26–28], it was therefore speculated that TREM2 might mediate the development of GBM radioresistance and immune escape through HMGB1/TLR4/Akt signaling pathway, siTREM2s were then transfected into GBM cells to verify this hypothesis. The results suggested that, besides TLR4 and its downstream molecule phospho-ERK1/2, TREM2 silencing significantly inhibited the expression of HMGB1, phospho-Akt and Akt (Fig. 4D). Moreover, TREM2 could bind to TLR4 and HMGB1 (Fig. 4E).

As secretory HMGB1 can bind to the membrane receptors of surrounding effector cells to exert biological effects, we further detected HMGB1 level in the culture supernatant of GBM^TREM2−KD^ cells, as shown in Fig. 4F, the secretion of HMGB1 from GBM^TREM2−KD^ cells were significantly decreased. It is well known that the extracellular HMGB1 is a typical damage associated molecular pattern (DAMP), the extracellular immunoglobulin domain of TREM2 can bind to DAMPs, and the interaction between TREM2 and HMGB1 had been confirmed as shown in Fig. 4E, so extracellular HMGB1 might be a ligand for TREM2 with the potential to regulate TREM2 expression. Subsequently, GBM cells were exposed to HMGB1 inhibitors with increasing concentrations, and the results demonstrated that HMGB1 inhibitor prominently downregulated the expression of TLR4, TREM2, phospho-Akt and Akt in a dose-dependent manner (Fig. 4G). These above results illustrated that there was a positive feedback loop between TREM2 and HMGB1, which exhibited cascade regulation of TLR4/Akt signaling pathway.

In addition, it has been found that HMGB1 participates in the formation of immunosuppressive TME and promotes M1-type macrophages transform into M2-type by interacting with TIM-3 [29], we further explored whether the conditioned culture medium of GBM^TREM2−KD^ cells affect the phenotype of microglia BV2. As shown in Fig. 4H, the conditioned culture medium of GL261^TREM2 − KD^ cells significantly inhibited the expression of TREM2 and TIM-3 and triggered the polarization of BV2 cells from M2-type to M1-type supported by the evidence of CD80 upregulation and CD206 downregulation. In addition, HMGB1 inhibitor could also clearly reduced the expression of TREM2, TIM-3 and CD206, stimulated CD80 expression on BV2 cells in a dose-dependent manner (Fig. 4I).

### The successfully synthesized A2-CM-NP/siTREM2/spam1 had splendid active targeting, homologous targeting and GBM accumulation

**Fig. 5:**
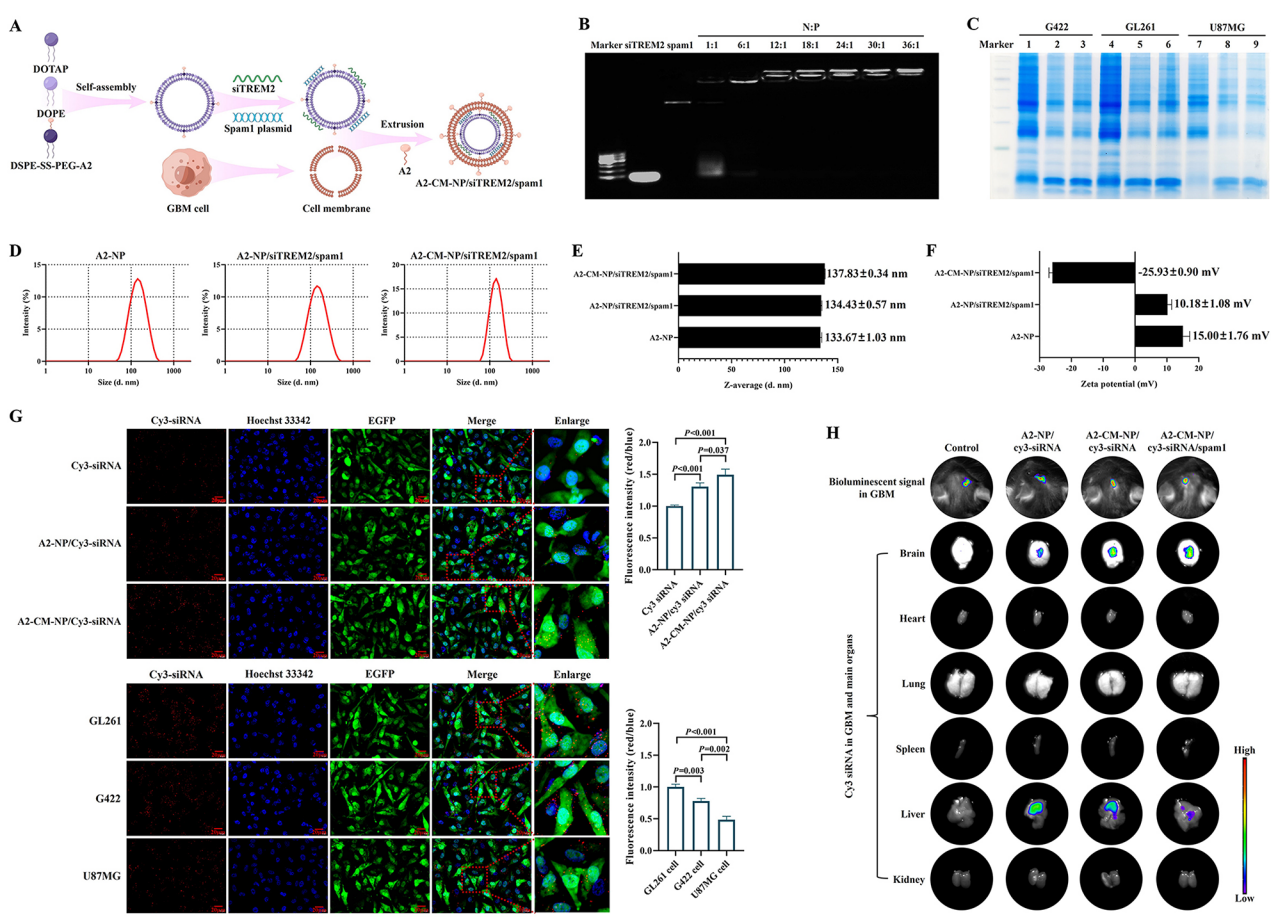
A2-CM-NP/siTREM2/spam1 was successfully constructed with superior active targeting, homologous targeting and in vivo biodistribution. (**A**) Schematic illustration of the synthesis process of A2-CM-NP/siTREM2/spam1; (**B**) Agarose gel eletrophoresis assay of the binding capacity of siTREM2 and spam1 plasmid to A2-NP at various N/P ratios; (**C**) SDS-PAGE analysis of proteins extracted from GBM cells (lanes 1, 4 and 7), GBM CM vesicles (lanes 2, 5 and 8), and A2-CM-NP/siTREM2/spam1 (lanes 3, 6 and 9) by Coomassie blue staining; The particle size distribution (**D**), statistical charts of particle sizes (**E**) and zeta-potentials (**F**) of A2-NP, A2-NP/siTREM2/spam1, and A2-CM-NP/siTREM2/spam1 were detected by dynamic light scattering, data are presented as mean ± SD, *n* = 3; (**G**) Up: representative fluorescent microscopy images and statistical chart of the red fluorescence intensity in GL261 cells cultured with cy3-siRNA, A2-NP/cy3-siRNA and A2-CM-NP/cy3-siRNA; down: GL261, G422, U87MG cells cultured with A2-CM-NP/cy3-siRNA synthesized with GL261 CM vesicles; red: cy3-siRNA, green: EGFP expressed in GBM cells, blue: cell nucleus stained with DAPI, scale bar = 20 μm. (**H**) In vivo bioluminescence imaging of mice after implanting luc-labeled GL261 cells for 7 days and ex vivo fluorescence imaging of the organs including brain, heart, liver, spleen, lungs and kidneys 24 h after the injection of PBS, A2-NP/cy3-siRNA, A2-CM-NP/cy3-siRNA, and A2-CM-NP/cy3-siRNA/spam1 via tail vein

In order to responsively release and efficiently concentrate the loaded siTREM2 in GBM region, GBM CM-biomimetic, GSH-responsive, A2-modificatory NPs named A2-CM-NP/siTREM2/spam1 were designed and synthesized according to the synthesis process shown in Fig. 5A, and the co-loaded spam1 plasmid could express hyaluronidase to degrade extracellular matrix. Because siTREM2 and spam1 plasmid were completely adsorbed when the N/P ratio was 12: 1 (Fig. 5B), this ratio was selected for subsequent experiments. As shown in Fig. 5C, the NPs possessed a similar protein profile as GBM CM vesicles, suggesting that GBM CM vesicles had been successfully coated onto the NPs, which was a key metric related to the quality of biomimetic NPs.

Moreover, the average size of A2-CM-NP/siTREM2/spam1 was 137.83 ± 0.34 nm, which satisfied passive targeting and was about 4 nm larger than the particle sizes of A2-NP (133.67 ± 1.03 nm) and A2-NP/siTREM2/spam1 (134.43 ± 0.57 nm) (Fig. 5D, E). The zeta potential of A2-NP was 15.00 ± 1.76 mV, which decreased to 10.18 ± 1.08 mV when loaded with siTREM2 and spam1 plasmid, and finally dropped to -25.93 ± 0.90 mV when coated with GBM CM vesicles (Fig. 5F). The above results manifested that A2-CM-NP/siTREM2/spam1 was successfully synthesized.

The active targeting of A2-CM-NP was then detected as indicated by the red fluorescence intensity of loaded cy3-siRNA in cells, and the result shown in Fig. 5G revealed that, compared with cy3-siRNA group, the intracellular red fluorescence intensity in A2-NP/cy3-siRNA group and A2-CM-NP/cy3-siRNA group were significantly increased, and the intracellular red fluorescence intensity of A2-CM-NP/cy3-siRNA group was the strongest, suggesting that A2-CM-NP had preferable active targeting and homologous targeting. The homologous targeting of A2-CM-NP synthesized with GL261 CM vesicles to GL261, G422 and U87MG cells were further evaluated, the result shown that GL261 group exhibited higher red fluorescence intensity than both G422 group and U87MG group, and the red fluorescence intensity in mouse cell line G422 was significantly stronger than that in human cell line U87MG (G422 group vs. U87MG group: *P* = 0.002, Fig. 5G), indicating the superior specific homologous targeting ability of the NPs.

The in vivo biodistribution and GBM accumulation of A2-CM-NP loading cy3-siRNA and spam1 plasmid was also investigated. The fluorescence intensity in GBM region of mice injected with A2-CM-NP/cy3-siRNA was significantly higher than that of A2-NP/cy3-siRNA group, and the strongest cy3 fluorescence intensity was observed in the GBM region of mice in A2-CM-NP/cy3-siRNA/spam1 group (Fig. 5H). In normal tissues, cy3 fluorescence was mainly distributed in liver, indicating that the NPs were principally metabolized and cleared through hepatic system, meanwhile, the fluorescence intensity in the liver of A2-NP/cy3-siRNA group, A2-CM-NP/cy3-siRNA group, and A2-CM-NP/cy3-siRNA/spam1 group successively decreased (Fig. 5H), suggesting that A2-CM-NP/cy3-siRNA/spam1 could rapidly penetrated BBB and degraded extracellular matrix barrier, thereby accelerating the accumulation of loaded drugs in the GBM region.

### Targeted TREM2 inhibition surpassingly improve the anti-GBM effect of radiotherapy and PD-1 inhibitor

**Fig. 6:**
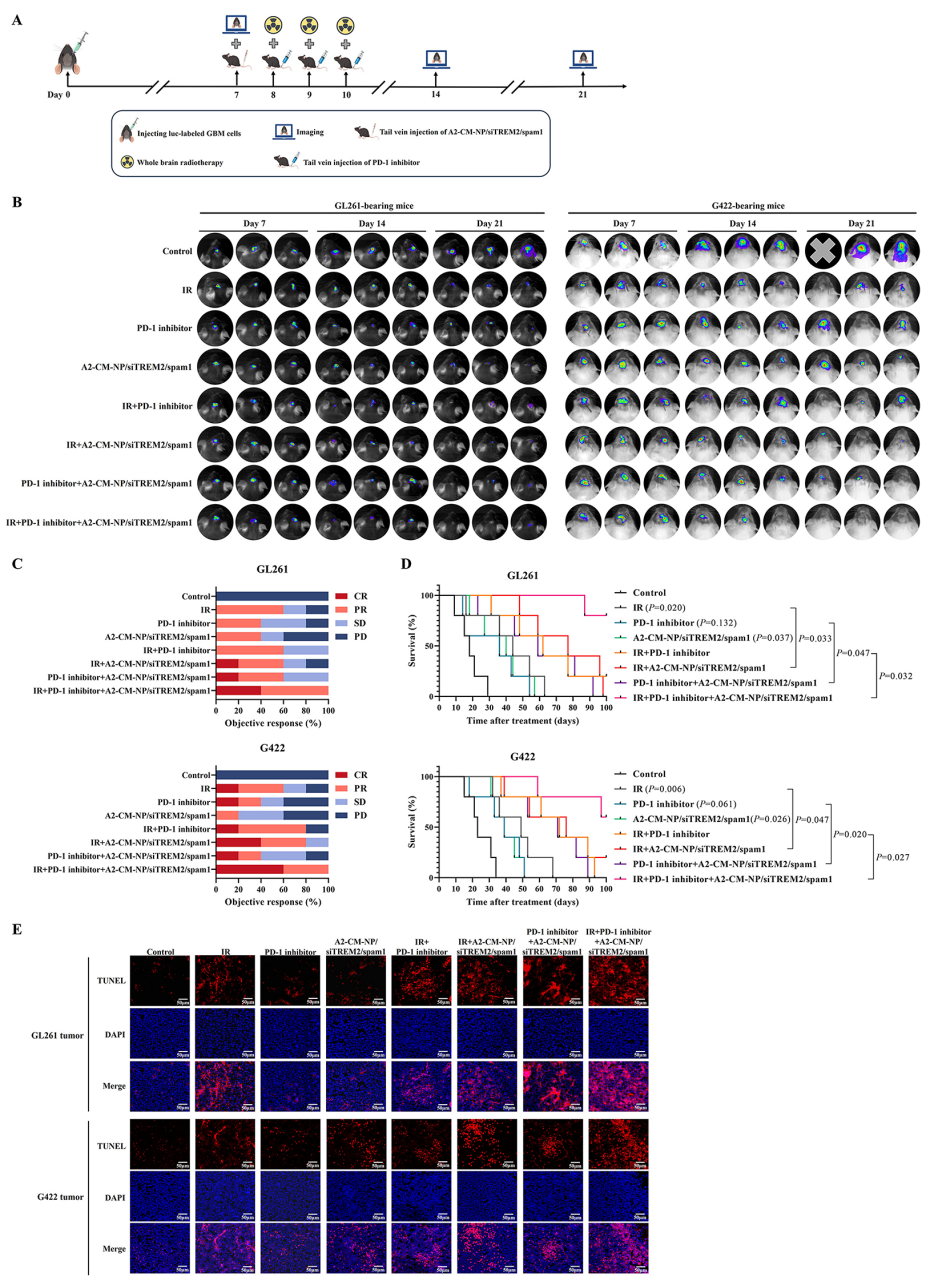
A2-CM-NP/siTREM2/spam1 prominently improved the therapeutic effect of radiotherapy and PD-1 inhibitor in intracranial GBM models. (**A**) Schematic illustration of the in vivo experimental workflow; (**B**) In vivo bioluminescent imaging of luc-labeled GL261-bearing mice and luc-labeled G422-bearing mice from each treatment group at different time; The short-term efficacy evaluation (**C**) and Kaplan − Meier survival curves (**D**) of orthotopic luc-labeled GL261-bearing mice and luc-labeled G422-bearing mice in all groups, *n* = 5; (**E**) The apoptosis rates in GL261 tissues and G422 tissues from different treatment groups were detected by TUNEL assay, and cell nucleus were stained with DAPI, scale bar = 50 μm

The anti-GBM efficacy of TREM2 inhibition was evaluated in mice intracranially inoculated with luc-labeled GBM cells according to the schematic illustration of treatment strategy shown in Fig. 6A. We discovered that radiotherapy, PD-1 inhibitor and A2-CM-NP/siTREM2/spam1 could inhibit GBM growth to a certain extent, the therapeutic strategies of radiotherapy combined with A2-CM-NP/siTREM2/spam1, PD-1 inhibitor combined with A2-CM-NP/siTREM2/spam1 evidently restrained the growth of GBM, and A2-CM-NP/siTREM2/spam1 combined with radiotherapy and PD-1 inhibitor exhibited the strongest tumoricidal activity (Fig. 6B). The objective response rate of IR + PD-1 inhibitor + A2-CM-NP/siTREM2/spam1 group was as high as 100%, and the complete response rate could surprisingly reach to 40%~60% (Fig. 6C). More importantly, compared with other treatments, IR + PD-1 inhibitor + A2-CM-NP/siTREM2/spam1 memorably prolonged OS (Fig. 6D).

In addition, the results of TUNEL assay suggested that the cell apoptosis rate of IR + PD-1 inhibitor + A2-CM-NP/siTREM2/spam1 group was more significant than other groups (Fig. 6E), which implied that TREM2 inhibition could further enhance the pro-apoptosis effect of radiotherapy and PD-1 inhibitor in GBM in vivo.

### TREM2 silencing increased and reprogramed the infiltrated T cells toward antitumor subpopulation in GBM microenvironment

**Fig. 7:**
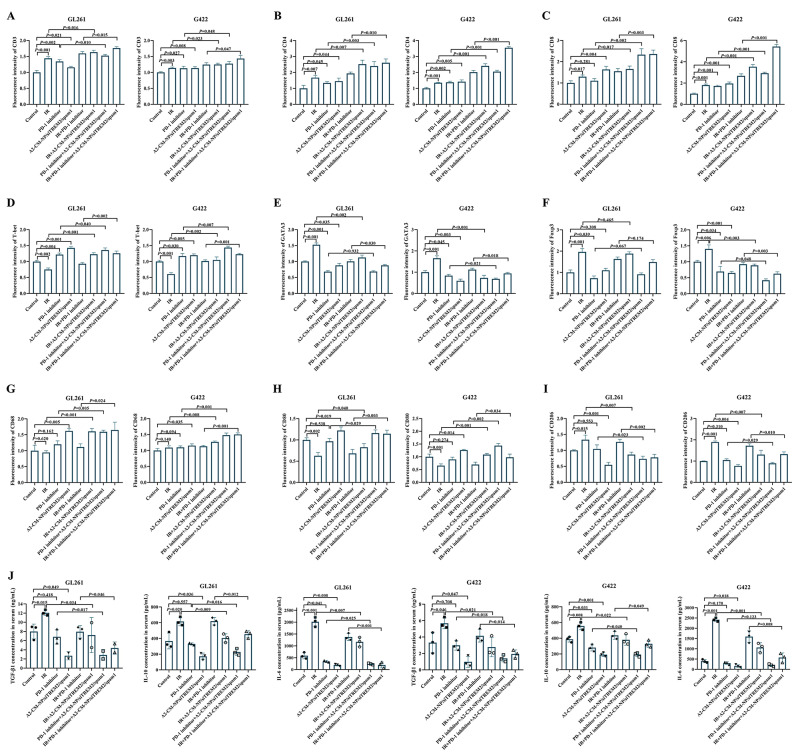
Inhibition of TREM2 reversed the immunosuppressive microenvironment of GBM. Statistical charts of the fluorescence intensity of CD3^+^ T cell (**A**), CD4^+^ T cells (**B**), CD8^+^ T cells (**C**), Th1 cells (T-bet^+^, **D**), Th2 cells (GATA3^+^, **E**), Treg cells (Foxp3^+^, **F**), macrophages (CD68^+^, **G**), M1-type macrophages (CD80^+^, **H**) and M2-type macrophages (CD206^+^, **I**) in GL261 tissues and G422 tissues; (**J**) The secretion levels of TGF-β1, IL-10 and IL-6 in peripheral blood of luc-labeled GL261-bearing mice and luc-labeled G422-bearing mice received different treatment were detected by ELISA assay, data are presented as mean ± SD, *n* = 3

Previous studies have reported that the rare T cell infiltration is one of the critical reasons for immunosuppressive GBM microenvironment, and the infiltrated T cells are mainly Th2 cells and Treg cells which promote GBM growth [15, 30, 31], so we then investigated whether the improved therapeutic outcomes of GBM with TREM2 inhibition was related to the changes of T cell infiltration and differentiation. As shown in Figs. 7A and S3, compared with control group, IR group, PD-1 inhibitor group and IR + PD-1 inhibitor group, the addition of A2-CM-NP/siTREM2/spam1 to them significantly facilitated T cells (CD3^+^) infiltrated to GBM region. Further analysis indicated that A2-CM-NP/siTREM2/spam1 could obviously increase the infiltration of CD4^+^T cells and CD8^+^T cells in GBM microenvironment (Figs. 7B, C and S3).

As CD4^+^T cells are highly heterogeneous, the Th2 subpopulation can counter the anti-tumor effect of Th1 subpopulation, and Treg cells possess the impact of inhibiting the proliferation of effector T cells and the secretion of immunosuppressive cytokines, thus instigating tumor cells to escape from immune surveillance [32]. We then investigated the effects of TREM2 inhibition on the infiltration of Th1, Th2 and Treg cells in GBM microenvironment. The results indicated that, A2-CM-NP/siTREM2/spam1 dramatically inhibited the recruitment of Th2 cells (GATA3^+^) and promoted the homing of Th1 cells (T-bet^+^), while radiotherapy significantly increased the number of Th2 cells and decreased the number of Th1 cells infiltrated in GBM microenvironment (Figs. 7D, E and S3). A2-CM-NP/siTREM2/spam1, to a certain extent, also reduced the infiltration of Treg cells (Foxp3^+^) with the evidence that obvious differences only appeared in the G422 mouse model, not GL261 mouse model (Figs. 7F and S3). These findings suggested that TREM2 inhibition could reprogram the increasing infiltrated T cells toward antitumor subpopulation.

### TREM2 inhibition induced macrophages repolarized to M1 type and reduced the secretion of pro-tumor cytokines

Since macrophages are thought to be the most important line of immune defense system of central nervous system [33], we further speculated TREM2 inhibition would affect their polarization. The results showed that radiotherapy could significantly increase the number of M2-type macrophages (CD206^+^CD68^+^) and decrease the number of M1-type macrophages (CD80^+^CD68^+^), while PD-1 inhibitor had little effect on the infiltration and polarization of macrophages; nevertheless, regardless of whether radiotherapy or PD-1 inhibitor was added, A2-CM-NP/siTREM2/spam1 prominently provoked the repolarization of macrophages from M2-type to M1-type (Figs. 7G–I and S3), signifying that TREM2 inhibition had the capacity of reversing the immunosuppressive microenvironment of GBM by domesticating macrophages to a favorable antitumor phenotype.

In addition to immune cells, cytokines are also important components of TME. The results of peripheral blood serum samples showed that, the secretion of TGF-β1, IL-10 and IL-6 were significantly stimulated in IR group, but decreased to varying degrees in PD-1 inhibitor group, and TREM2 inhibition group elicited the most memorable change with simultaneous reduction of all the detected cytokines (Fig. 7J).

### A2-CM-NP/siTREM2/spam1 had low toxic and side effects in vivo

The toxic and side effects of A2-CM-NP/siTREM2/spam1 were evaluated by observing the body weight changes of GBM-bearing mice and the morphological changes of main organs (heart, liver, spleen, lungs and kidneys). As shown in Fig. S4 A–C, A2-CM-NP/siTREM2/spam1 did not obviously decrease the body weight of GBM-bearing mice, instead, the body weight of mice in IR + PD-1 inhibitor + A2-CM-NP/siTREM2/spam1 group showed a gradual increase trend after treatment (Fig. S4 C). The results of H&E staining (Fig. S4 D, E) indicated that A2-CM-NP/siTREM2/spam1 did not cause obvious morphological damage to normal organs, implying this therapeutic strategy could not only effectively reverse the radioresistance and immune escape of GBM, but also had good safety in vivo.

## Discussion

Radioresistance and immune escape are the key reasons for unsatisfactory therapeutic effects of most malignancies, especially GBM. GBM is prone to radioresistance because of its stronger and more active DNA damage repair system than other tumors [6], and its severe immunosuppression of TME often leads to the failure of many clinical trials on ICIs [13]. Currently, the well-studied mechanisms of improving radiosensitivity are to increase the primary DNA damage induced by radiotherapy and inhibit the repair ability of sub-lethal and lethal damages of tumor cells, and the most important lethal DNA damage is DNA double strand break, which further actives DNA-PKcs, KU70 and KU80 to complete DNA damage repair [34]. In this study, we discovered that the D_q_ values of GBM^TREM2−KD^ cells, which represented the ability of cells to repair sub-lethal damage, were lower than corresponding GBM-NC cells (Table S3), and TREM2 silencing decreased the expression of DNA-PKcs, KU70 and KU80 in GBM cells (Fig. 2H), suggesting TREM2 inhibition significantly and directly enhanced the radiosensitivity of GBM through interfering with DNA double strand break repair pathway.

It has been demonstrated that only a limited fraction of patients with GBM benefitted from existing ICIs, which means exploring other immune regulatory targets is urgently needed. In 2023, Yan M. et al. demonstrated that Siglec-9, an immune-checkpoint molecule on macrophages, could be targeted to enhance the therapeutic efficacy of anti-PD-1/PD-L1 for GBM treatment [35]. Ni X. et al. identified galectin-9/TIM-3 as a viable target against GBM, especially PTEN-deficient GBM through inhibiting macrophage M2 polarization [36]. However, previous studies on immune targets for GBM mainly focused on immune cells, this study aimed to inhibit TREM2 expression in GBM cells themselves, so as to directly enhanced the radiosensitivity of GBM cells while affecting key immune cells in the GBM microenvironment by regulating the secretion of cytokines. Here, we found that TREM2 was significantly positively correlated with HAVCR2 gene encoding TIM-3 (Fig. 3E), which was identified as a potential candidate for cancer immunotherapy due to its roles in mediating the effective termination of Th1 cells, inducing the exhaustion of T cells and enhancing the immunosuppressive function of Treg cells [37–40]. In vitro, the conditioned culture supernatant of GBM^TREM2−KD^ cells significantly decreased TIM-3 expression of macrophages (Fig. 4H). More importantly, the in vivo results disclosed that TREM2 inhibition visibly reduced the infiltration of Th2 and Treg cells and increased the infiltration of Th1 cells in GBM microenvironment (Fig. 7).

In terms of molecular mechanisms, we innovatively found that there is a positive feedback loop between TREM2 and HMGB1, TREM2 silencing could decrease the expression of HMGB1 in cells and the secretion of HMGB1 into the extracellular space, which acted as a DAMP and bound to the extracellular domain of TREM2, and HMGB1 inhibitor could further inhibit TREM2 expression (Fig. 4D–G). HMGB1 exhibits several functions in the treatment resistance and immunosuppression through interacting with DNA damage repair pathway and regulating signaling pathways including TLR4 and PI3K/AKT [41, 42]. Moreover, TLR4 activation is related to the immune escape of cancer cells, inducing the resistance of cancer cells against cytotoxic T lymphocytes [43], and the activation of TLR4/Akt signaling promotes the development of cancer [44]. In this study, we found that TREM2 silencing by siTREM2 transfection significantly downregulated the activation of TLR4/Akt signaling, which could be further decreased by the inhibition of HMGB1/TREM2 positive feedback loop (Fig. 4D–G).

The treatment pattern of combining radiotherapy and immunotherapy was proposed in 2005 [45], and then completely subverted the therapeutic strategy of stage III unresectable non-small cell lung cancer based on the announcement of the PACIFIC study [46, 47]. However, subsequent study suggested that, compared with sequential radiotherapy and ICIs, concurrent radiotherapy and ICIs could significantly improve the prognosis of patients with immunologically “cold” tumors with lower toxicity and better tolerance [48]. Therefore, the therapeutic strategy of concurrent radiotherapy and ICI instead of sequential radiotherapy and ICI was selected in vivo, and the encouraging results revealed that TREM2 inhibition prominently improved the anti-GBM effects of radiotherapy and PD-1 inhibitor (Fig. 6).

However, there are still some limitations in this study. For example, the in vivo experiments were not conducted with knockout mouse, the level of released tumor-specific antigens during therapy was not detected, and whether this combination therapy increased the risk of cognitive impairment in GBM-bearing mice was also not assessed. Up to now, TREM2 has been developed as a potential target for Alzheimer’s disease, solid tumors and other diseases, although no drug targeting TREM2 has been approved, many pharmaceutical companies have laid out the research pipeline of targeting TREM2, and our findings will also further accelerate the clinical translation and application of therapies targeting TREM2.

## Conclusions

In this study, we found that TREM2 was a regulator in accelerating the radioresistance and immune escape of GBM through participating in DNA damage repair and forming a positive feedback loop with HMGB1 to cascade the activation of TLR4/Akt signaling.

In order to efficiently delivery siTREM2 to GBM cells, GBM CM-biomimetic, GSH-responsive and A2-modificatory NPs with satisfied passive targeting, active targeting and homologous targeting were synthesized and used for in vivo study. Targeted TREM2 inhibition exhibited remarkable anti-GBM therapeutic effect through promoting the infiltration of Th1 cells and CD8^+^T cells, reducing the infiltration of Th2 cells and Treg cells, repolarizing macrophages to M1-type, and decreasing the secretion of pro-tumor and immunosuppressive cytokines. Therefore, targeting TREM2 therapy is a promising avenue for optimizing radiotherapy and immunotherapy to improve the prognosis of GBM patients.

### Electronic supplementary material

Below is the link to the electronic supplementary material.

Supplementary Material 1

Supplementary Material 2

Supplementary Material 3

Supplementary Material 4

## Data Availability

The datasets used and analyzed during the current study are available from the corresponding author on reasonable request.

## Abbreviations

A2: Angiopep-2
Akt: Protein Kinase B
BBB: Blood-brain Barrier
BED: Bioequivalent Dose
CM: Cell Membrane
Co-IP: Co-immunoprecipitation
DAMP: Damage Associated Molecular Pattern
DAPI: 4’,6-diamidino-2-phenylindole
ddH_2_O: Double Distilled Water
DEGs: Differentially Expressed Genes
DEPC: Diethyl Pyrocarbonate
DMEM: Dulbecco Modified Essential Medium
DNA-PKcs: DNA-dependent Protein Kinase Catalytic Subunit
DOPE: Dioleoylphosphatidylethanolamine
DOTAP: 1,2-dioleoyl-3-trimethylammonium propane
DSPE: 1,2-distearoyl-sn-glycero-3-phosphoethanolamine
EGFP: Enhanced Green Fluorescent Protein
ELISA: Enzyme-linked Immunosorbent Assay
FBS: Fetal Bovine Serum
GBM: Glioblastoma
GO: Gene Ontology
GSH: Glutathione
H&E: Hematoxylin and Eosin
HMGB1: High Mobility Group Box 1
ICI: Immune Checkpoint Inhibitor
IFN-γ: Interferon-gamma
IL-6: Interleukin 6
IL-10: Interleukin 10
IR: Irradiation
KD: Knockdown
KEGG: Kyoto Encyclopedia of Genes and Genomes
Luc: Luciferase
Mal: Maleimide
NC: Negative Control
NP: Nanoparticle
OS: Overall Survival
PARP1: Poly ADP-ribose Polymerase 1
PBS: Phosphate Buffer Solution
PD-1: Programmed Cell Death Protein 1
PI3K: Phosphatidylinositol 3 Kinase
PVDF: Polyvinylidene Fluoride
SD: Standard Deviation
SDS-PAGE: Sodium Dodecyl Sulphate-Polyacrylamide Gel Electrophoresis
SER: Sensitizing Enhancement Ratio
SF2: Surviving Fraction at 2 Gy
shRNA: Short Hairpin RNA
siRNA: Small Interfering RNA
siTREM2: Small Interfering RNA Targeting TREM2
SS: Disulfide Bond
sTREM2: soluble TREM2
STRING: Search Tool for Recurring Instances of Neighboring Genes
TCGA: The Cancer Genome Atlas Program
TGF-β1: Transforming Growth Factor-β1
Th1: Type 1 Helper T Cell
TIM-3: T cell Immunoglobulin Domain and Mucin Domain-3
TIMER 2.0: Tumor Immune Estimation Resource 2.0
TLR4: Toll-like Receptor 4
TREM2: Triggering Receptor Expressed on Myeloid Cells-2
TREM2: TREM2 Knockdown
Treg: Regulatory T cell
TME: Tumor Microenvironment
TUNEL: Terminal Deoxynucleotidyl Transferase-mediated dUTP-biotin Nick End Labeling
