# Supplementary material for: HMGB1/TREM2 positive feedback loop drives the development of radioresistance and immune escape of glioblastoma by regulating TLR4/Akt signaling
Source: J Transl Med. 2024 Jul 29;22:688. doi: 10.1186/s12967-024-05489-w (PMC11287841; doi:10.1186/s12967-024-05489-w)
Supplement: Supplementary file 1 — Supplementary Material 1 [file 12967_2024_5489_MOESM1_ESM.docx]

**Table S1.** The sequences of siTREM2 and siNC.

| **siRNAs** | **Sense (5’-3’)** | **Anti-sense (5’-3’)** |
| --- | --- | --- |
| siTREM2#1 | GUCAUGUACUUAUGACGCCUUTT | AAGGCGUCAUAAGUACAUGACTT |
| siTREM2#2 | GAGCACAGUCAUCGCAGAUGATT | UCAUCUGCGAUGACUGUGCUCTT |
| siTREM2#3 | AGAUGCUGGGCACCAACUUCATT | UGAAGUUGGUGCCCAGCAUCUTT |
| siTREM2#4 | CCUUGCUGGAACCGUCACCAUTT | AUGGUGACGGUUCCAGCAAGGTT |
| siTREM2#5 | GACCCUCUAGAUGACCAAGAUTT | AUCUUGGUCAUCUAGAGGGUCTT |
| siTREM2#6 | AGCACCUCCAGGAAUCAAGAGTT | CUCUUGAUUCCUGGAGGUGCUTT |
| siNC | UUCUCCGAACGUGUCACGUTT | ACGUGACACGUUCGGAGAATT |
