# Supplementary material for: HMGB1/TREM2 positive feedback loop drives the development of radioresistance and immune escape of glioblastoma by regulating TLR4/Akt signaling
Source: J Transl Med. 2024 Jul 29;22:688. doi: 10.1186/s12967-024-05489-w (PMC11287841; doi:10.1186/s12967-024-05489-w)
Supplement: Supplementary file 2 — Supplementary Material 2 [file 12967_2024_5489_MOESM2_ESM.docx]

**Table S2.** Radiosensitive parameters of U87MG-R and GL261-R cells.

| **Cell lines** | **R^2^** | **D0** | **N** | **Dq** | **D37** | **SF2** | **α** | **β** | **α/β** | **SER_D0_** |
| --- | --- | --- | --- | --- | --- | --- | --- | --- | --- | --- |
| U87MG | 0.982 | 2.344 | 1.709 | 1.256 | 3.600 | 0.605 | 0.202 | 0.024 | 8.417 | 0.819 |
| U87MG-R | 0.940 | 2.863 | 2.882 | 3.030 | 5.893 | 0.841 | 0.040 | 0.024 | 1.667 | - |
| GL261 | 0.972 | 2.484 | 1.769 | 1.417 | 3.901 | 0.656 | 0.187 | 0.020 | 9.350 | 0.805 |
| GL261-R | 0.925 | 3.087 | 2.729 | 3.099 | 6.186 | 0.866 | 0.045 | 0.021 | 2.143 | - |
