# Supplementary material for: HMGB1/TREM2 positive feedback loop drives the development of radioresistance and immune escape of glioblastoma by regulating TLR4/Akt signaling
Source: J Transl Med. 2024 Jul 29;22:688. doi: 10.1186/s12967-024-05489-w (PMC11287841; doi:10.1186/s12967-024-05489-w)
Supplement: Supplementary file 3 — Supplementary Material 3 [file 12967_2024_5489_MOESM3_ESM.docx]

**Table S3.** Radiosensitive parameters of TREM2-knockdown glioblastoma cells.

| **Cell lines** | **R^2^** | **D0** | **N** | **Dq** | **D37** | **SF2** | **α** | **β** | **α/β** | **SER_D0_** |
| --- | --- | --- | --- | --- | --- | --- | --- | --- | --- | --- |
| U87MG-NC | 0.993 | 2.995 | 1.849 | 1.841 | 4.836 | 0.749 | 0.137 | 0.016 | 8.563 | 1.223 |
| U87MG^TREM2-KD^ | 0.980 | 2.448 | 1.639 | 1.210 | 3.658 | 0.621 | 0.216 | 0.019 | 11.368 | - |
| GL261-NC | 0.994 | 2.870 | 1.919 | 1.871 | 4.741 | 0.716 | 0.123 | 0.020 | 6.15 | 1.244 |
| GL261^TREM2-KD^ | 0.943 | 2.307 | 1.748 | 1.288 | 3.595 | 0.604 | 0.197 | 0.025 | 7.88 | - |
| G422-NC | 0.986 | 2.822 | 1.907 | 1.822 | 4.644 | 0.708 | 0.128 | 0.021 | 6.095 | 1.310 |
| G422^TREM2-KD^ | 0.974 | 2.155 | 1.610 | 1.026 | 3.181 | 0.557 | 0.257 | 0.022 | 11.682 | - |
