# Supplementary material for: HMGB1/TREM2 positive feedback loop drives the development of radioresistance and immune escape of glioblastoma by regulating TLR4/Akt signaling
Source: J Transl Med. 2024 Jul 29;22:688. doi: 10.1186/s12967-024-05489-w (PMC11287841; doi:10.1186/s12967-024-05489-w)
Supplement: Supplementary file 4 — Supplementary Material 4 [file 12967_2024_5489_MOESM4_ESM.docx]

**Table S4.** Top 20 genes associated with the radioresistance and immune escape of GBM.

|  | **Top 20 genes** | |
| --- | --- | --- |
| **Radioresistance** | | **EGFR** **STAT3** **TP53** AKT1 FOXM1 **PTEN** HIF1A ATM **VEGFA** **IL6** NOTCH1 NFE2L2 CCND1 TERT MTOR MET YAP1 **TGFB1** **MYC HMGB1** |
| **Immune escape** | | FOXP3 **STAT3** TNFRSF14 TLR4 **TP53** **MYC** CD274 CD40 TNFAIP3 SOCS1 **IL6** **EGFR** **HMGB1** **VEGFA** ERBB2 SMAD3 **PTEN** IDO1 **TGFB1** CXCR4 |
